# Supplementary material for: The impact of repeated rapid test strategies on the effectiveness of at-home antiviral treatments for SARS-CoV-2
Source: Nat Commun. 2022 Sep 8;13:5283. doi: 10.1038/s41467-022-32640-2 (PMC9453717; doi:10.1038/s41467-022-32640-2)
Supplement: Supplementary file 1 — Supplementary Information [file 41467_2022_32640_MOESM1_ESM.pdf]

Supporting Information for "The impact of repeated rapid test strategies on the effectiveness of at-home antiviral treatments for SARS-CoV-2"
(Tigist F Menkir and Christl A Donnelly)

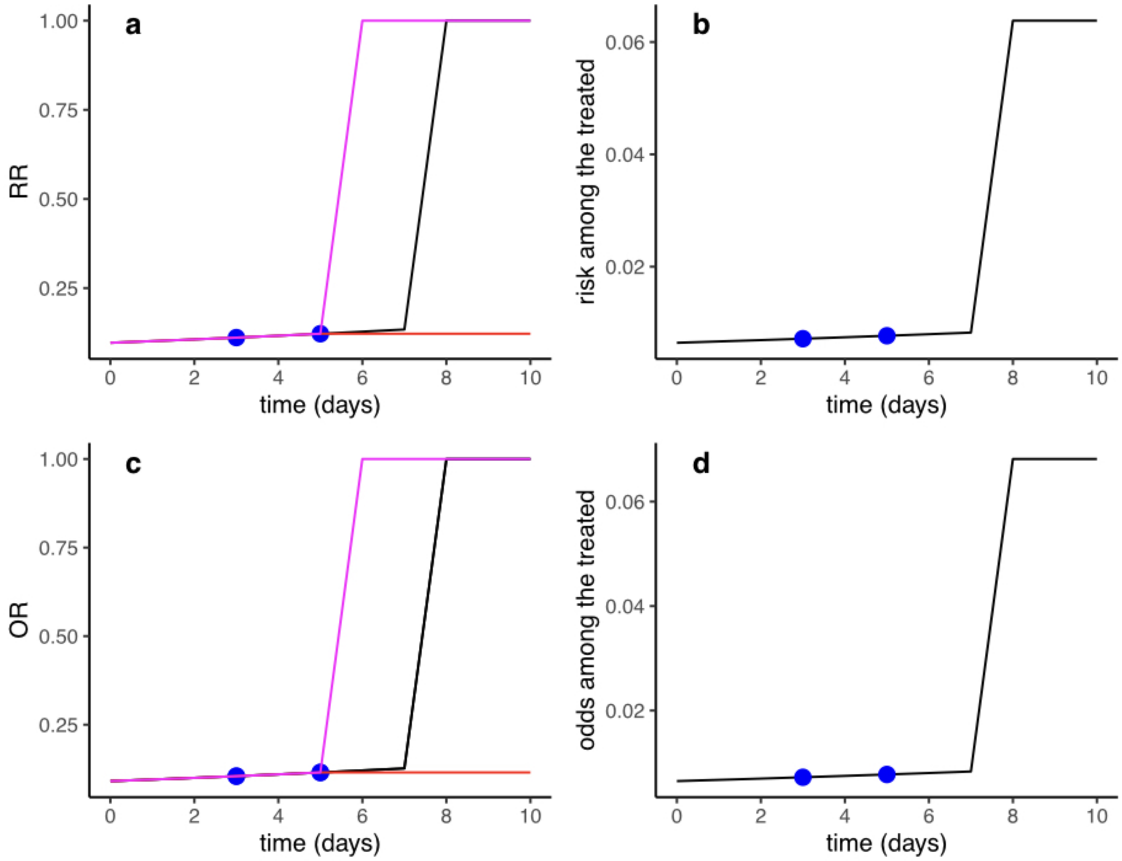

Supplementary Figure S1 Estimated hospitalization risk ratios (RR), relative to the patient population untreated with nirmatrelvir, risks among the treated, odds ratios (OR), relative to the patient population untreated with nirmatrelvir, and odds. (A) Estimated relative risks of hospitalization as a function of time of treatment relative to symptom onset (B) Estimated risks of hospitalization among subjects treated with nirmatrelvir as a function of time of treatment relative to symptom onset (C) Estimated odds ratio of hospitalization as a function of time of treatment relative to symptom onset (D) Estimated odds of hospitalization among subjects treated with nirmatrelvir as a function of time of treatment relative to symptom onset. Blue points indicate observed RRs (A), risks among the treated (B), ORs (C) and odds among the treated (D) at three and five days of symptom onset as reported in the EPIC-HR press release.[1] In panels A and C, the black lines capture estimates under our main scenario, the pink lines capture estimates under the fast decline to zero efficacy scenario, and the red lines capture estimates under our efficacy preserved scenario.

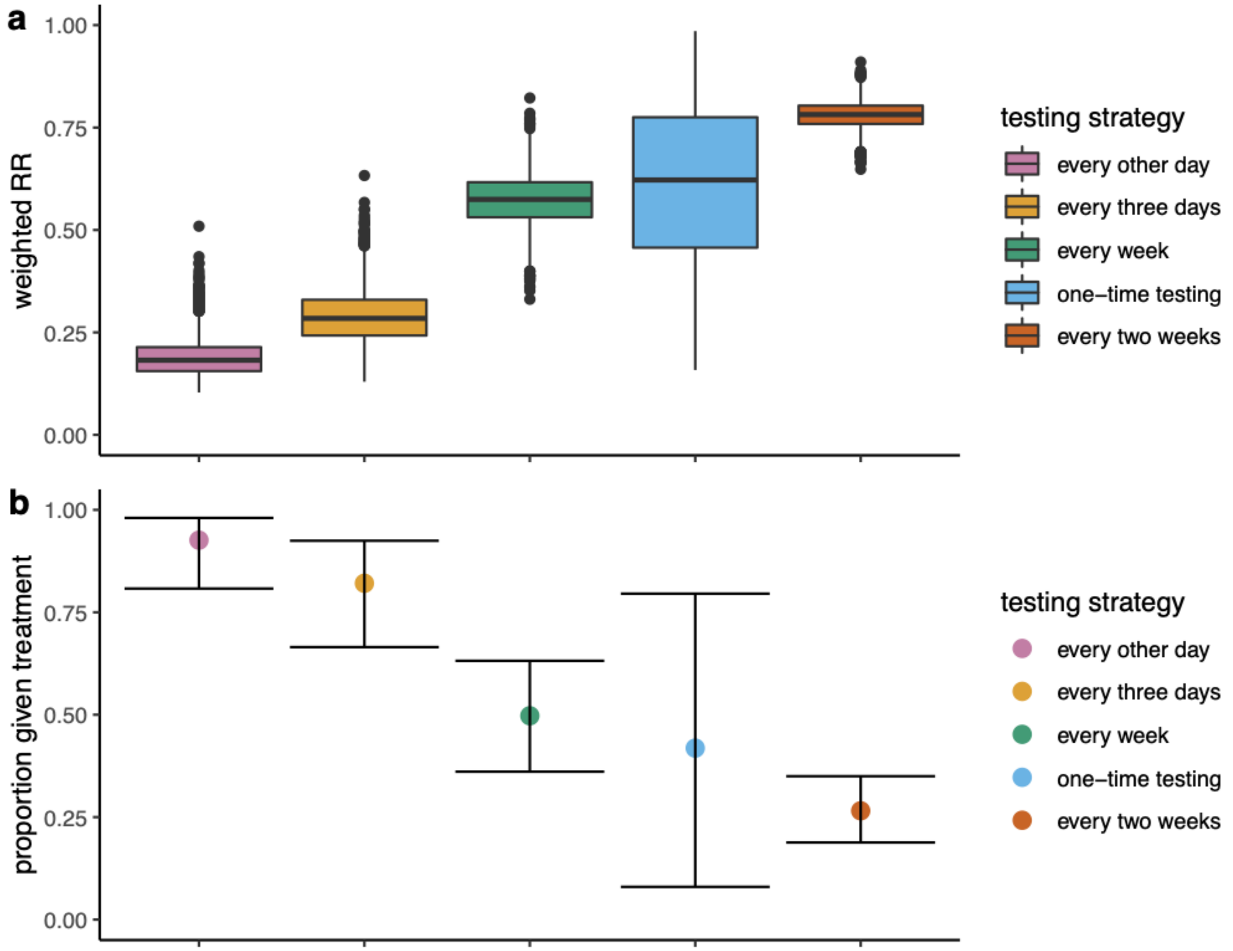

Supplementary Figure S2 Estimated weighted hospitalization risk ratios (weighted RR), relative to the patient population untreated with nirmatrelvir, and estimated proportions given treatment, by testing strategy (under the shorter incubation scenario). The weighting of RRs reflects the likelihood of testing positive and therefore being treated with nirmatrelvir x days after becoming infected. (A) Distribution of estimated weighted risk ratios of hospitalization by testing strategy: every other day (pink), every three days (orange), every week (green), one-time testing (blue) and every two weeks (dark orange). Medians are marked by solid horizontal lines, each box includes the full interquartile range, and plotted points are those which extend beyond the upper/lower quartile +/- 1.5*interquartile range (B) Estimated median proportions given treatment by testing strategy (including the one-time post-symptom-onset testing strategy) with 95% CIs. In all cases no positive-test-to-treatment delay and full treatment and test coverage were assumed. n=4000 MCMC samples, each consisting of positivity estimates up to 30 days since infection[2]

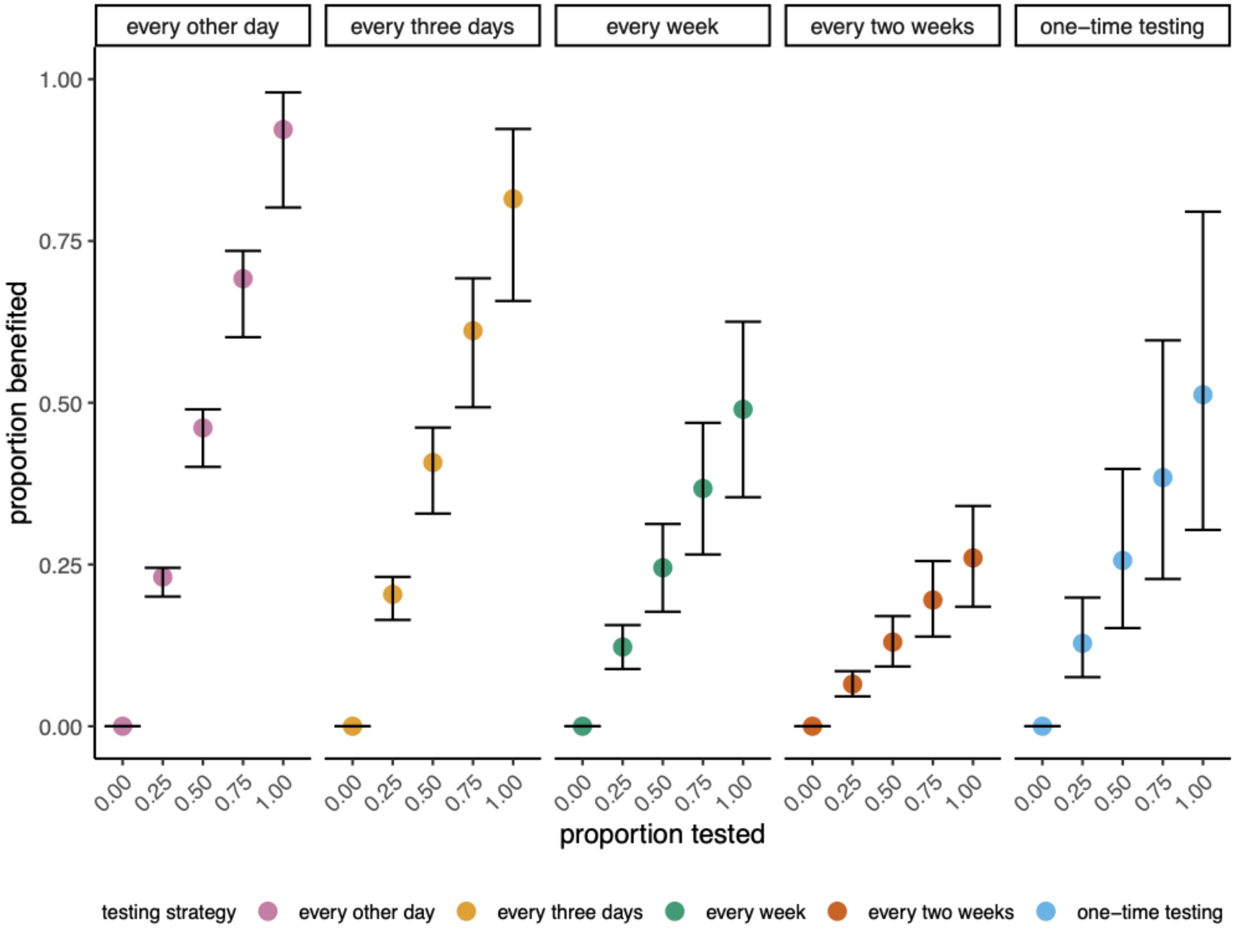

Supplementary Figure S3 Sensitivity of estimated proportions benefiting from treatment to the proportion who tests, across testing strategies. Estimated median proportions who benefit from treatment, with corresponding 95% CIs, as a function of proportion of the population who tests. Each vertical panel corresponds to a testing strategy (every other day (pink), every three days (orange), every week (green), every two weeks (dark orange), one-time testing (blue)). n=4000 MCMC samples, each consisting of positivity estimates up to 30 days since infection[2]

Supplementary Table S1. Estimated weighted risk ratios of hospitalization, relative to the patient population untreated with nirmatrelvir, and estimated proportions given treatment and proportion benefited, by testing strategy, under the incubation period distribution assumed in our main analysis (baseline) and in our shorter incubation period scenario. The weighting of RRs reflects the likelihood of testing positive and therefore being treated with nirmatrelvir x days after becoming infected.

Testing Strategy	Median RR (95% CI) [baseline incubation period scenario]	Median RR (95% CI) [shorter incubation period scenario]	Median proportion benefited (95 % CI) [baseline incubation period scenario]	Median proportion benefited (95 % CI) [shorter incubation period scenario]
**Every other day**	0.168 (0.114-0.277)	0.182 (0.122-0.299)	0.922 (0.802-0.980)	0.913 (0.784-0.977)
**Every three days**	0.265 (0.164-0.408)	0.284 (0.175-0.431)	0.815 (0.657-0.923)	0.800 (0.638-0.917)
**Once every week**	0.559 (0.429-0.682)	0.575 (0.447-0.695)	0.490 (0.354-0.625)	0.478 (0.342-0.616)
**Once every two weeks**	0.766 (0.690-0.834)	0.782 (0.712-0.844)	0.260 (0.185-0.340)	0.245 (0.175-0.320)
**One-time testing**	0.537 (0.271-0.726)	0.622 (0.272-0.928)	0.512 (0.303-0.795)	0.418 (0.0798-0.795)

References

1. Pfizer: Media relations. Pfizer Announces Additional Phase 2/3 Study Results Confirming Robust Efficacy of Novel COVID-19 Oral Antiviral Treatment Candidate in Reducing Risk of Hospitalization or Death [Internet]. 2021. Available from: https://www.pfizer.com/news/press-release/press-release-detail/pfizer-announces-additional-phase-23-study-results
2. Hellewell J, Russell TW, The SAFER Investigators and Field Study Team, et al. Estimating the effectiveness of routine asymptomatic PCR testing at different frequencies for the detection of SARS-CoV-2 infections [Internet]. Epidemiology; 2020
