# Supplementary material for: The impact of repeated rapid test strategies on the effectiveness of at-home antiviral treatments for SARS-CoV-2
Source: Nat Commun. 2022 Sep 8;13:5283. doi: 10.1038/s41467-022-32640-2 (PMC9453717; doi:10.1038/s41467-022-32640-2)
Supplement: Supplementary file 3 — Reporting Summary [file 41467_2022_32640_MOESM3_ESM.pdf]

nature portfolio

Corresponding author(s): Christl A Donnelly

Last updated by author(s): May 11, 2022

Reporting Summary

Nature Portfolio wishes to improve the reproducibility of the work that we publish. This form provides structure for consistency and transparency in reporting. For further information on Nature Portfolio policies, see our Editorial Policies and the Editorial Policy Checklist.

Statistics

For all statistical analyses, confirm that the following items are present in the figure legend, table legend, main text, or Methods section.

n/a	Confirmed	
☐	☒	The exact sample size (n) for each experimental group/condition, given as a discrete number and unit of measurement
☒	☐	A statement on whether measurements were taken from distinct samples or whether the same sample was measured repeatedly
☒	☐	The statistical test(s) used AND whether they are one- or two-sided<br>*Only common tests should be described solely by name; describe more complex techniques in the Methods section.*
☐	☒	A description of all covariates tested
☐	☒	A description of any assumptions or corrections, such as tests of normality and adjustment for multiple comparisons
☐	☒	A full description of the statistical parameters including central tendency (e.g. means) or other basic estimates (e.g. regression coefficient) AND variation (e.g. standard deviation) or associated estimates of uncertainty (e.g. confidence intervals)
☒	☐	For null hypothesis testing, the test statistic (e.g. F, t, r) with confidence intervals, effect sizes, degrees of freedom and P value noted<br>*Give P values as exact values whenever suitable.*
☒	☐	For Bayesian analysis, information on the choice of priors and Markov chain Monte Carlo settings
☒	☐	For hierarchical and complex designs, identification of the appropriate level for tests and full reporting of outcomes
☒	☐	Estimates of effect sizes (e.g. Cohen's d, Pearson's r), indicating how they were calculated

Our web collection on statistics for biologists contains articles on many of the points above.

Software and code

Policy information about availability of computer code

Data collection	All analyses were conducted using R (version 4.0.3 and Rstudio (1.2.1355)
Data analysis	LFT positivity data was sourced using the open source code provided in https://github.com/cmmid/pcr-profile, specifically "aux_funcs.R", "pcr_breakpoint.stan", and relevant segments from "run_analysis.R", based on the following paper by Hellewell et al.: https://bmcmedicine.biomedcentral.com/track/pdf/10.1186/s12916-021-01982-x.pdf. Pfizer drug efficacy data was obtained from the latest EPIC-HR summary release at https://www.pfizer.com/news/press-release/press-release-detail/pfizer-announces-additional-phase-23-study-results. We provide code to 1) estimate the relative risks, risk, odds ratios, and odds of hospitalization in treatment and placebo groups using the counts reported in the latest EPIC-HR summary report, 2) estimate weighted risk ratios of hospitalization and output the results for our main scenario under each of the testing strategies we consider using the full positivity data extracted from the Hellewell et al. analysis, 3) estimate weighted risk ratios of hospitalization and output the results for each of the additional drug efficacy sensitivity analyses we consider, 4) estimate weighted risk ratios of hospitalization and output the results using the summary positivity data extracted from the Hellewell et al. analysis under a range of assumptions about drug coverage and test-to-treatment delays, 5) estimate proportions offered or benefiting from treatment as a function of the proportion of the population who tests, and 5) generate figures to summarize the aforementioned output

For manuscripts utilizing custom algorithms or software that are central to the research but not yet described in published literature, software must be made available to editors and reviewers. We strongly encourage code deposition in a community repository (e.g. GitHub). See the Nature Portfolio guidelines for submitting code & software for further information.

Data

Policy information about availability of data

All manuscripts must include a data availability statement. This statement should provide the following information, where applicable:

- Accession codes, unique identifiers, or web links for publicly available datasets
- A description of any restrictions on data availability
- For clinical datasets or third party data, please ensure that the statement adheres to our policy

All code and input data can be accessed here: https://github.com/goshgondar2018/LFT_treatment_analysis

Field-specific reporting

Please select the one below that is the best fit for your research. If you are not sure, read the appropriate sections before making your selection.

☒ Life sciences ☐ Behavioural & social sciences ☐ Ecological, evolutionary & environmental sciences

For a reference copy of the document with all sections, see nature.com/documents/nr-reporting-summary-flat.pdf

Life sciences study design

All studies must disclose on these points even when the disclosure is negative.

Sample size	No sample size calculations were performed. Sample sizes were restricted to that of the positivity estimates provided in the Hellewell et al. analysis
Data exclusions	No data were excluded from our analyses
Replication	No experimental trials were run. Our findings were replicated upon additional runs.
Randomization	Randomization or patient allocation was not relevant to our study as we directly used estimates of group-level (i.e. for the treatment and placebo groups) hospitalization risks as reported in the Pfizer EPIC-HR trial.
Blinding	Blinding was not relevant to our study, as described above

Reporting for specific materials, systems and methods

We require information from authors about some types of materials, experimental systems and methods used in many studies. Here, indicate whether each material, system or method listed is relevant to your study. If you are not sure if a list item applies to your research, read the appropriate section before selecting a response.

Materials & experimental systems

n/a	Involved in the study	
☒	☐	Antibodies
☒	☐	Eukaryotic cell lines
☒	☐	Palaeontology and archaeology
☒	☐	Animals and other organisms
☒	☐	Human research participants
☒	☐	Clinical data
☒	☐	Dual use research of concern

Methods

n/a	Involved in the study	
☒	☐	ChIP-seq
☒	☐	Flow cytometry
☒	☐	MRI-based neuroimaging
